# Marked Enhancement of Roll-Off Frequency in FeCoN Synthetic Antiferromagnetic Films Deposited by Oblique Incidence

**DOI:** 10.3390/ma12142328

**Published:** 2019-07-22

**Authors:** Luran Zhang, Dandan Gao, Huan Liu, Jiyang Xie, Wanbiao Hu

**Affiliations:** School of Materials Science and Engineering, Yunnan University, Kunming 650091, China

**Keywords:** magnetic materials, high frequency properties, synthetic antiferromagnetic, thin films

## Abstract

A series of FeCoN films were successfully deposited on glass substrates in a magnetron sputtering system. Using oblique incidence method and FeCoN/Ru/FeCoN synthetic antiferromagnetic (SAF) structure, two additional anisotropies energy were introduced: oblique incidence anisotropy and exchange anisotropy energy, which marked enhancement of the effective magnetic anisotropy (Hk). The increment of Hk results in a significant improvement in the roll-off frequency of these films. The roll-off frequency of FeCoN/Ru/FeCoN films with SAF structure can reach up to 8.6 GHz. A feasible approach to conveniently controlling Hk of soft magnetic thin films by using oblique deposition and SAF structure can further improve their properties for the potential applications in the high frequency region.

## 1. Introduction

With the fast development of communication and information technologies, more challenges lie in front of radio-frequency and microwave device designers. Magnetic films with high roll-off frequency (*f*_r_) on radio-frequency and microwave applications are required [1,2]. Nowadays, in many devices, the operating frequencies have reached gigahertz bands [3,4]. The *f*_r_ of magnetic thin films used in these devices should be very high, ideally beyond 5 GHz or even 10 GHz. The *f*_r_ is significantly influenced by the effective magnetic anisotropy (*H*_k_) and saturation magnetization (*M*_s_) of the magnetic film. According to the Landau–Lifshitz–Gilbert (LLG) equation, *f*_r_ is defined as

(1) $$f_{r}=\frac{\gamma }{2\pi }\mu_{0}\sqrt{M_{s}H_{k}}$$

where γ is the gyromagnetic factor and *μ*_0_ is the permeability of vacuum [5].

For obtaining the higher range of ferromagnetic resonance frequency, high saturation magnetization materials with a large effective anisotropy field should be considered. As we know, FeCo alloy with 60–70 at.% Fe shows the highest saturation magnetization (up to 24 kGs) among the metal magnetic materials. But FeCo thin films with isotropic magnetic properties are not applicable for high frequency applications. Many efforts have been adopted to increase the *H*_k_ of FeCo thin films, such as applying an induced magnetic field during film deposition [6], depositing on prestressed substrates [7] or flexible substrates [8], field annealing [9], stress inducing [10], oblique deposition [11], micro strip patterning [12], introducing gradient of the Hf concentration [13,14] and using an organic semiconductors underlayer [15]. However, it is still a big challenge getting a very large *H*_k_ in soft magnetic films and enhancing their *f*_r_ beyond 5 GHz. Besides, FeCo is difficult to achieve good soft magnetic properties because the FeCo alloy shows a large saturation magnetostriction coefficient (around (40–65) × 10^−6^). FeCoN thin film is attracting more and more attentions since its good soft magnetic property and high potential in high-frequency applications [16,17,18,19]. 

In this work, FeCoN single film and FeCoN/Ru/FeCoN synthetic antiferromagnetic (SAF) structure films are produced by using a direct current magnetron sputtering equipment with different oblique angles. Oblique deposition and SAF structure can obviously enhance the *H*_k_ and *f*_r_ of FeCoN films. The films prepared at an increasing oblique angle show an enhancement of the *H*_k_ of FeCoN single and SAF structure films. Magnetic properties, antiferromagnetic behavior and high-frequency performance of the films are discussed.

## 2. Methods

**Figure 1:**
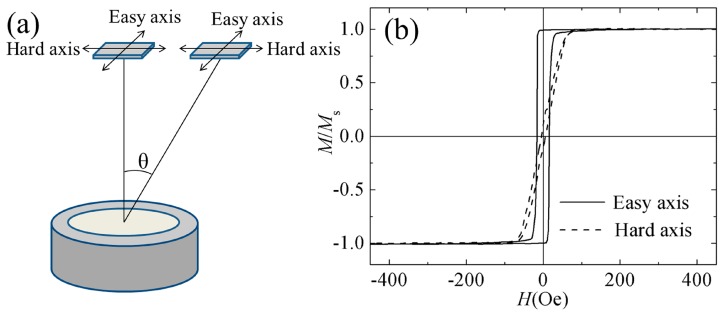
(**a**)Schematic drawing of the sputtering arrangement. (**b**) Hysteresis loops of single film (*f*_N_ = 5%) with oblique angle 0°.

FeCoN (40 nm) film and FeCoN (20 nm)/Ru (0.45 nm)/FeCoN (20 nm) SAF structure films were prepared on glass substrates (10 × 10 mm^2^) at room temperature by using a direct current magnetron sputtering equipment (Kurt J. Lesker, Jefferson Hills, USA) with a 3-inch Fe_65_Co_35_ target and a 3-inch Ru target. The schematic diagram of the sputtering arrangement was shown in Figure 1a. The distance between substrate and target was 12 cm. Films were deposited at an oblique angle (*θ*) ranging from 0° to 26°. A static magnetic field about 600 Oe was applied in the substrate plane which was defined as the easy axis (EA) direction. The sputtering power of FeCoN layer was 50 W and that of Ru interlayer was fixed at 15 W. The low pressure with 1 × 10^−7^ Torr in the sputtering system was applied. A mixture of Ar and N_2_ (with a flow rate—*f*_N_ = N_2_/(Ar + N_2_) × 100%—of 5%), was applied during the sputtering process of FeCoN layers. The sputtering gas during Ru layer deposition was pure Ar. The depositing pressure was 5.0 mTorr. A vibrating sample magnetometer (VSM YP07-VSM-130, Zhongxi, Beijing, China) was utilized to measure the magnetic properties of the films at room temperature. The film electric resistivity at room temperature was measured using the standard four-point measurement technique (ST2263, JGDZ, Suzhou, China). A vector network analyzer (PNA E8363B, Keysight, Santa Rosa, USA) via the microstrip method [20] was introduced to measure the permeability spectra. The composition of films was detected using an energy dispersive spectrometer (EDS, FEI, Hillsboro, USA). The Fe concentration of all films were fixed at 66 ± 1%, which changed little with an oblique angle. 

## 3. Results and Discussion

**Figure 2:**
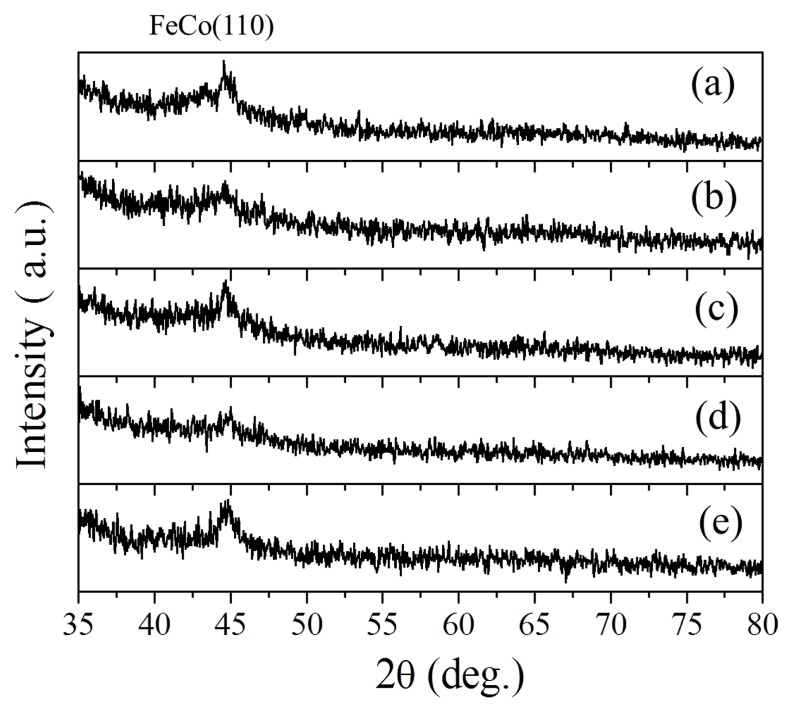
XRD pattern of FeCoN single films deposited at oblique angle with 0° (**a**), 13° (**b**), 26° (**c**) and FeCoN SAF films deposited at oblique angle with 0° (**d**) and 26° (**e**), respectively.

A 5% N_2_ flow ratio was chosen for the FeCoN single layer and SAF structure films because of the high saturation magnetization, low coercivity, and high electric resistivity. The in-plane hysteresis loops of FeCoN single film (*f*_N_ = 5%) are shown in Figure 1b. The FeCoN film’s saturation magnetization 4π*M_s_* is about 21.6 kGs. The EA coercivity *H_ce_*, hard axis (HA) coercivity *H_ch_* and the effective anisotropy field *H*_k_ of FeCoN film are 15.5 Oe, 5 Oe and 55 Oe respectively. The electric resistivity is 250 μΩ·cm. The XRD pattern of FeCoN film without oblique angle is shown in Figure 2a. It can be seen that only FeCo (110) peak presents, which means that the FeCoN film shows 110 texture. The FeCo (110) peak is wide and weak, which means that the FeCoN film shows a nanocrystalline structure.

**Figure 3:**
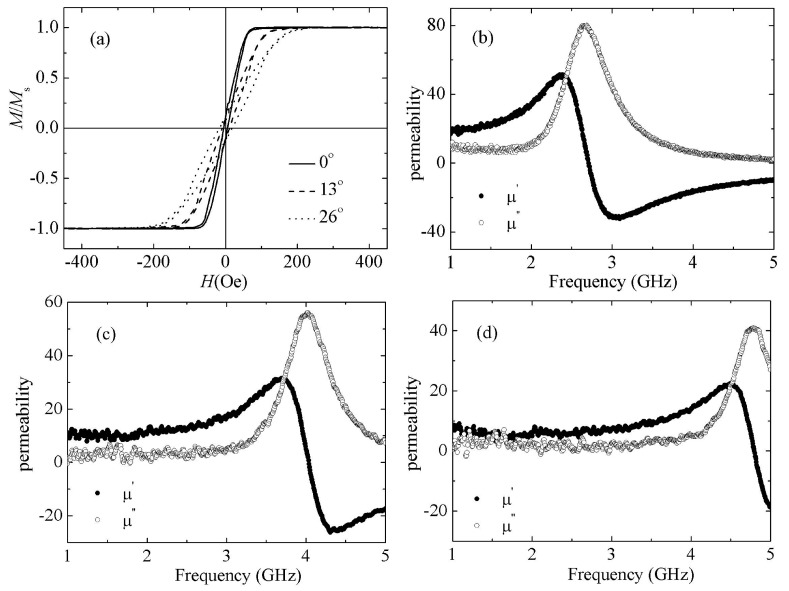
Hard axis hysteresis loops (**a**) and high frequency performance for FeCoN single films deposited at oblique angle with 0° (**b**), 13° (**c**) and 26° (**d**), respectively.

FeCoN single layer films are deposited at different oblique angle in order to increase the *H*_k_. The XRD patterns of FeCoN films with oblique angle at 13°, 26° are shown in Figure 2b,c, respectively. They are not much different to FeCoN film without an oblique angle, only FeCo (110) peak appear. Figure 3a shows the hard axis hysteresis loops of the FeCoN single films deposited at oblique angles of 0°, 13° and 26°. It is found that the anisotropy increases with an increasing oblique angle. The coercivity of HA are slightly increased under larger oblique angle. The coercivity of EA also increase under larger oblique angle, but much smaller than that of HA. The saturation magnetization is found to be not dependent on position. The *H*_k_ of films are improved gradually with the increment of oblique angle. The *H*_k_ of films prepared with oblique angles at 0°, 13°, 26° are 55 Oe, 105 Oe and 160 Oe, respectively. These results reveal that the *H*_k_ of these films rely on the sputtering angle at deposition. The change of effective anisotropy is attributed to an additional anisotropy energy *K*^oblique^ where *K*^total^ = *K*^induced^ + *K*^oblique^ with *H*_k_ = 2*K*^total^/*M*_s_. According to the Equation (1), the increment of *H*_k_ results in the improvement of the roll-off frequency. As shown in Figure 3b–d, the roll-off frequency of FeCoN single films deposited at oblique angles at 0°, 13° and 26° are 2.6, 4.1 and 4.7 GHz, respectively.

**Figure 4:**
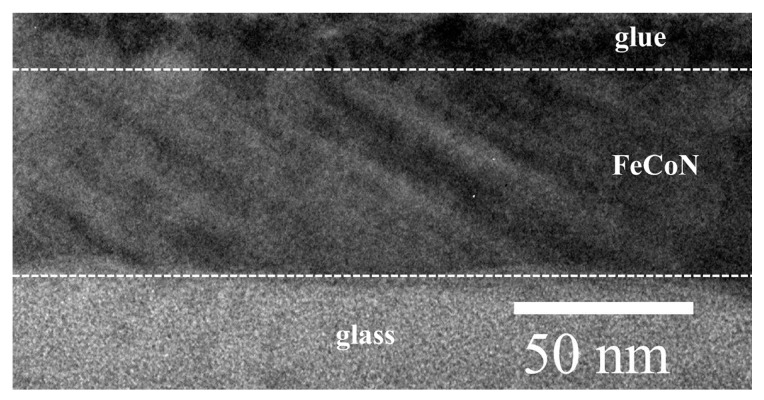
Cross section TEM image of FeCoN single layer film deposited at oblique angle with 26°.

Figure 4 shows the TEM image of FeCoN single layer film deposited at oblique angle with 26°. It can be seen that the oblique columnar growth of FeCoN. The oblique incidence anisotropy is caused by the columnar growth morphology with the increase of oblique angle [21,22].

**Figure 5:**
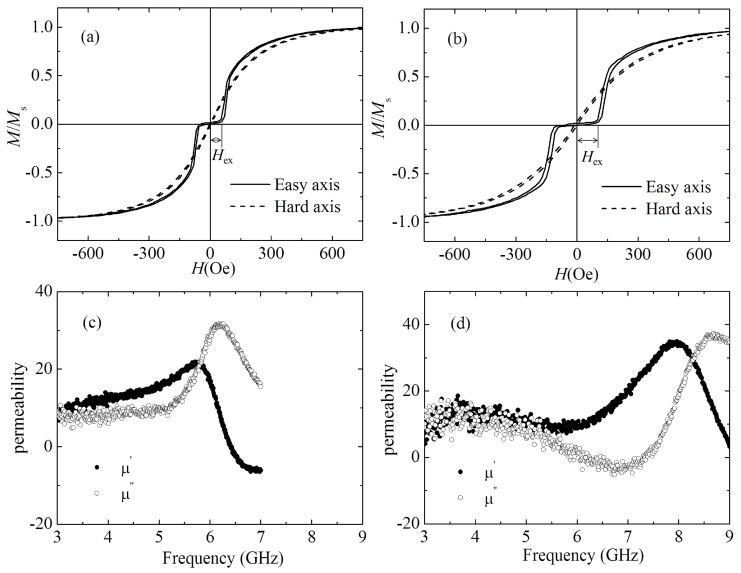
Hysteresis loops (**a**,**b**) and high frequency performance (**c**,**d**) for glass/FeCoN (20 nm)/Ru (0.45 nm)/FeCoN (20 nm) films deposited at oblique angle 0° and 26°, respectively.

To introduce exchange anisotropy, the FeCoN SAF structure films are prepared. In the SAF structure, two ferromagnetic (FM) layers separated by a nonmagnetic (NM) metallic spacer layer have a Ruderman–Kittel–Kasuya–Yosida (RKKY) interaction which is indirect interaction through interlayer exchange coupling [23]. Depending on the thickness, the NM layer can mediate either a ferromagnetic or antiferromagnetic exchange coupling between two FM layers [24]. The XRD patterns of FeCoN SAF structure films with oblique angle at 0° and 26° are shown in Figure 2d,e, respectively. It can be seen that only weak FeCo (110) peak appears. Figure 5a shows the hysteresis loops of FeCoN SAF structure film (glass/FeCoN (20 nm)/Ru (0.45 nm)/FeCoN (20 nm)) with the oblique angle of 0°. The SAF structure film shows distinct separated hysteresis in the EA while the HA loop shows a slanted loop. In the case of a stacked film with interlayer coupling, the magnetization is almost canceled out around the zero field, which means the magnetic moments alignment of the top and bottom magnetic layers are antiparallel. With the increase of the external field, the magnetic moment cancels each other at first. When the external field increases to a certain extent, the magnetic moment reverses. When the external field increases further, the magnetic moments tend to saturate through a rotation-magnetized process. The exchange field (*H*_ex_) of glass/FeCoN (20 nm)/Ru (0.45 nm)/FeCoN (20 nm)) without oblique angle is 80 Oe. Here, *H*_ex_ is the smallest field rotating the magnetization in one of two ferromagnetic layers from the antiparallel alignment. Because of the exchange coupling between the bottom and top magnetic layers, there is an additional anisotropy energy (*K*^exchange^) in FeCoN/Ru/FeCoN film, and *K*^total^ = *K*^induced^ + *K*^exchange^. *H*_k_ = 2*K*^total^/*M*_s_, so the FeCoN sandwich films’ *H*_k_ are larger than that of FeCoN single film. Figure 5c shows the frequency spectrum of FeCoN SAF structure film with the oblique angle of 0°. Compared to the FeCoN single layer film, the roll-off frequency increases from 2.6 to 6.2 GHz. Figure 5b,d shows the hysteresis loops and the high frequency performance of FeCoN SAF structure film with the oblique angle of 26°, respectively. The roll-off frequency of this film drastically increase to 8.6 GHz, which is larger than that of the film prepared without an oblique angle. In this film, *K*^total^ does not only include *K*^induced^, *K*^exchange^, but also includes *K*^oblique^, so *K*^total^ = *K*^induced^ + *K*^exchange^ + *K*^oblique^. Therefore, this film’s *H*_k_ and the roll-off frequency are larger than that of the film which deposited without an oblique angle. It’s should be noticed that the *H*_ex_ of FeCoN sandwich film with oblique angle of 26° is 138 Oe, which is larger than that of the film with 0°. It means FeCoN sandwich film with oblique angle of 26° has larger exchange coupling energy than the film with 0° although they have same structure.

## 4. Conclusion

FeCoN single films and FeCoN/Ru/FeCoN SAF structure films were produced by utilizing a direct current magnetron sputtering equipment under the oblique angles from 0° to 26°. The films deposited with the increase of oblique angle showed an improvement of the *H*_k_ of the FeCoN single and SAF structure films. By using the oblique incidence method and FeCoN/Ru/FeCoN SAF structure, two additional anisotropies energy *K*^oblique^ and *K*^exchange^ are introduced, which can obviously enhance the *H*_k_. The increase of *H*_k_ results in a significant improvement of the roll-off frequency. The roll-off frequency of FeCoN films were tuned from 2.6 to 8.6 GHz. For the application of soft magnetic thin films with high frequency, controlling the magnitude of *H*_k_ is essential. Compared to other methods for increasing the *H*_k_ of FeCo thin films, such as depositing on prestressed substrates [7] or flexible substrates [8], micro strip patterning [12], introduces gradient of the Hf concentration [14,15] and using an organic semiconductors underlayer [16], our method is quite simple, more manageable and can get much more higher roll-off frequency. In this work, a feasible approach to conveniently controlling *H*_k_ of FeCoN thin films by using oblique deposition and SAF structure can further improve their properties for the potential applications in the high frequency region.
